# The nature of triple-negative breast cancer classification and antitumoral strategies

**DOI:** 10.5808/GI.2020.18.4.e35

**Published:** 2020-12-22

**Authors:** Songmi Kim, Dong Hee Kim, Wooseok Lee, Yong-Moon Lee, Song-Yi Choi, Kyudong Han

**Affiliations:** 1 Department of Nanobiomedical Science, Dankook University, Cheonan 31116, Korea; 2 Department of Anesthesiology and Pain Management, Dankook University Hospital, Cheonan 31116, Korea; 3 Center for Bio-Medical Engineering Core Facility, Dankook University, Cheonan 31116, Korea; 4 Department of Pathology, Dankook University School of Medicine, Cheonan 31116, Korea; 5 Department of Pathology, Chungnam National University School of Medicine, Daejeon 35015, Korea; 6 Department of Microbiology, Dankook University, Cheonan 31116, Korea

**Keywords:** classification, gene expression, immune checkpoint blockade, microbiome, subtype, triple-negative breast cancer

## Abstract

Identifying the patterns of gene expression in breast cancers is essential to understanding their pathophysiology and developing anticancer drugs. Breast cancer is a heterogeneous disease with different subtypes determined by distinct biological features. Luminal breast cancer is characterized by a relatively high expression of estrogen receptor (ER) and progesterone receptor (PR) genes, which are expressed in breast luminal cells. In ~25% of invasive breast cancers, human epidermal growth factor receptor 2 (HER2) is overexpressed; these cancers are categorized as the HER2 type. Triple-negative breast cancer (TNBC), in which the cancer cells do not express ER/PR or HER2, shows highly aggressive clinical outcomes. TNBC can be further classified into specific subtypes according to genomic mutations and cancer immunogenicity. Herein, we discuss the brief history of TNBC classification and its implications for promising treatments.

## Introduction

Breast cancer is one of the most common cancers in women and is a leading cause of cancer-related deaths. In 2018, ~266,000 women were expected to be diagnosed with invasive breast cancer in the United States, and 40,920 women were expected to die from it [1]. Triple-negative breast cancer (TNBC), which accounts for 15% of all breast cancers, is characterized by the lack of estrogen receptor (ER), progesterone receptor (PR), and human epidermal growth factor receptor 2 (HER2) expression, which means that TNBC patients do not benefit from hormonal therapy or trastuzumab, which targets HER2 [2-4]. TNBC generally occurs in younger women and compared to other types of breast cancer, it shows a high histologic grade, a high propensity to metastasize to distant organs, and a poor outcome with a high recurrence rate after adjuvant therapy, which mainly consists of systemic cytotoxic chemotherapy [4]. Therefore, a significant challenge is to identify new targets and associated biomarkers for its treatment. Compared to other breast cancer types, TNBC is characterized by a higher mutational load, which makes the tumor immunogenic and amenable to immunotherapeutic treatment [4]. Thanks to the promising results of immunotherapy in some cancers such as malignant melanoma and non-small cell lung cancer [5,6], several clinical trials are currently assessing immunotherapeutic approaches in TNBC patients [4,7-11]. The U.S. Food and Drug Administration (FDA) approval of nanoparticle albumin-bound paclitaxel (nab-paclitaxel) combined with atezolizumab in 2019 presented an innovative therapeutic development for TNBC patients. In this review, we discuss a brief history of TNBC classification based on gene expression patterns, as well as promising anticancer strategies for TNBC.

## What Is TNBC?

In 1999, a molecular classification of breast cancer was first proposed by the National Cancer Institute (NCI) [12]. In 2000, Perou et al. [13] introduced a classification of breast cancer into four types: luminal, basal-like, HER2, and normal breast-like. The mammary epithelium consists of two layers: luminal cells and basal (myoepithelial) cells. The term “luminal” refers to the part containing mammary lobules and ductal structures, which are the major targets of estrogen and progesterone and mature to produce milk. In contrast, the term “basal” denotes another part of the mammary epithelium that supports the lobular and ductal structures. Sorlie et al. [14] further subdivided the luminal type into luminal A and B. The luminal A type demonstrated relatively high expression of the luminal epithelial gene cluster, including ER, GATA3, X-box binding protein 1, trefoil factor 3, hepatocyte nuclear factor 3α, FOXA1, and LIV-1. In contrast, the luminal B type showed high expression of proliferation-related genes and low to moderate expression of luminal-specific genes [14]. In a following study, breast cancer was classified into three major types based on the expression or absence of ER, PR, and HER2, namely ER+/PR+/HER2‒ (luminal A type), HER2+, and TNBC. The HER2+ type can be further divided into two subtypes: ER+/PR+/HER2+ (luminal B type) and ER‒/PR‒/HER2+ (HER2+ type) [15]. TNBC often occurs in under 40-year-old women and has a mortality rate of 40% within the first 5 years after diagnosis, a median survival time after metastasis of only 13.3 months, and a recurrence rate after surgery of 25%. Therefore, gene expression profiling analysis has begun to enable a deeper understanding of this disastrous disease, which is not sensitive to hormone therapy or HER2 treatment [2].

## The Classification Begins!

Classification and analysis are milestones for scientific understanding. Now, it is TNBC’s turn. In 2011, Lehmann et al. [9] classified TNBC into six subtypes using 587 TNBC patients based on transcriptome profiling data as follows:

(1) The basal-like 1 subtype is involved in cell cycle and cell division pathways and shows overexpression of the *AURKA, AURKB, BIRC5, BUB1, CENPA, CENPF, CCNA2, MYC, NRAS, PRC1, PLK1*, and *TTK* genes. In addition, the expression of DNA repair (ATR/BRCA pathway)–related genes, such as *CHEK1, FANCA, FANCG, RAD54BP, RAD51, NBN, EXO1, MSH2, MCM10, RAD21*, and *MDC1*, is significantly increased.

(2) The basal-like 2 subtype is associated with increased expression of growth factor signaling pathways, including the EGF, NGF, MET, Wnt/β-catenin, and IGF1R pathways.

(3) The immunomodulatory subtype (IM subtype) shows high activation of immune signaling pathways (CTLA4, natural killer (NK)-cell, Th1/Th2, NFKB, TNF, T-cell, JAK/STAT, ATR/BRCA), and cytokine signaling pathways, such as the interleukin (IL)-12 and IL7 pathways.

(4) The mesenchymal subtype (M subtype) exhibits significantly lower expression levels in the immune signal transduction pathway, unlike the IM subtype. The M subtype also shows profound activation of cell migration-related signaling pathways, extracellular matrix receptor interaction pathways, and cell differentiation pathways, such as the Wnt pathway, anaplastic lymphoma kinase pathway, and transforming growth factor (TGF)-β pathway. These molecular changes result in sarcomatous morphological features.

(5) The mesenchymal stem-like subtype (MSL subtype) features high expression of stemness-related pathways, including the inositol phosphate metabolism pathway, G-protein-coupled receptor pathway, and calcium signaling pathway. In addition, the MSL subtype displays high expression of angiogenesis pathways such as *KDR, TEK, TIE1*, and *EPAS1*, but very low expression of the proliferative pathway. Moreover, this subtype is accompanied by high expression of stem cell markers (*ABCA8, PROCR, ENG, ALDHA1, PER1, ABCB1, TERT2IP*, and *BCL2*) and mesenchymal stem cell-specific markers (*BMP2, ENG, ITGV, KDR, NGFR, NTSE, PDGFR, THY1*, and *VCAM1*).

(6) The luminal androgen receptor subtype (LAR subtype) displays high expression of hormonal-related signaling pathways, including steroid synthesis, porphyrin metabolism, and androgen/estrogen metabolism.

Subsequently, Burstein et al. [16] proposed four molecular subgroups using gene expression profiling of 198 TNBC cases as follows.

(1) The LAR subgroup is characterized by gene expression for hormone-related signaling pathways, including prolactin signaling and estrogen/androgen metabolism. The tumors within this subgroup show androgen receptor, ER, prolactin, and ErbB4 signaling, but are ERα-negative by immunohistochemistry (IHC) staining. *ESR1* and other estrogen-regulated genes such as *PGR, FOXA, XBP1*, and *GATA3* are expressed. This group demonstrates ER activation despite belonging to the category of ER-negative tumors by IHC, suggesting that traditional anti-estrogen therapies and anti-androgen therapies might be useful [9].

(2) The mesenchymal subgroup shows activation of pathways related to the complement system, prothrombin activation, coagulation system, leukocyte extravasation signaling, and hepatic stellate cell activation signaling. In addition, this subgroup shows down-regulation of several signaling pathways, including cell cycle, mismatch repair, and hereditary breast carcinoma signaling pathways. In general, genes exclusive to osteocytes (*OGN*) and adipocytes (*ADIPOQ*, and *PLIN1*), and insulin-like growth factors (*IGF-1*) are highly expressed in this subgroup.

(3) The basal-like immune-suppressed (BLIS) subgroup is characterized by down-regulation of B cell, T cell, and NK cell immune-regulating pathways and cytokine pathways. Activation of the cell cycle and DNA repair-related signaling pathways has also been identified in this subgroup.

(4) The basal-like immune-activated (BLIA) subgroup, unlike the BLIS subgroup, shows up-regulation of B cell, T cell, and NK cell immune-regulating pathways. Additionally, the expression levels of STAT genes are elevated, and STAT transcription factor-mediated pathways are highly activated in this subgroup.

Recently, Liu et al. [17] proposed four new subtypes after a classification analysis of the gene expression profile combined with mRNAs and long noncoding RNAs in 165 TNBC samples, as follows.

(1) The IM subtype has high expression levels of genes related to innate immune response T-cell co-stimulation and the immune response, such as *CCR2, CXCL13, CXCL11, CD1C, CXCL10*, and *CCL5*.

(2) The LAR subtype, despite being ER-negative on IHC staining, shows activation of the ER signaling pathway. Steroid biosynthesis, porphyrin metabolism, androgen/estrogen metabolism, and peroxisome proliferator-activated receptor signaling pathways are highly activated in this subtype.

(3) The mesenchymal-like subtype is enriched with various gene ontology category members and signaling pathways, such as extracellular matrix-receptor interactions, gap junctions, TGF-β, growth factor pathways, and the adipocytokine signaling pathway. Contrariwise, the mesenchymal-like subtype shows down-regulation of cell proliferation-related genes (cell division process, mitotic cell cycle, mitotic prometaphase, and mitosis).

(4) The BLIS subtype is highly enriched in cell division and cell cycle-related signaling pathways, including DNA replication, DNA repair, mitotic cell cycle, mitotic prometaphase, and the M phase of the mitotic cell cycle. The BLIS subtype has high expression of proliferation-related genes, such as *CENPF, BUB1*, and *PRC1*, but this subtype is characterized by significant down-regulation of immune cell signaling pathways, immune response, and complement activation processes. The TNBC subtypes discussed above are summarized in Table 1.

## Immune Checkpoint Blockade Therapy in Certain TNBC Subtypes

Targeting cytotoxic T-lymphocyte-associated antigen 4 (CTLA-4), programmed cell death protein 1 (PD-1), and its ligand (PD-L1) has revolutionized cancer treatment. CTLA-4 signaling is more prevalent in lymph nodes, and PD-1/PD-L1 is involved in multiple processes in the tumor microenvironment, such as suppression of T-cell responses and T-cell anergy. Blockade of PD-1/PD-L1 can significantly induce T-cell proliferation and activity, generating antitumor immune responses [6,18]. Thanks to this, immune checkpoint blockade (ICB) therapies have become a mainstay of treatment. According to Karn et al. [19], TNBC can be divided into immune-rich or immune-poor subtypes based on metagenes for a high lymphocyte infiltration (major histocompatibility complex class II) gene signature and low inflammation markers, such as interleukin-8 and vascular endothelial growth factor. Tumors with high immune gene expression had a better prognosis due to strict immunosurveillance and were associated with a low level of clonal heterogeneity, somatic copy number alteration level, mutations, and neoantigen load [19,20]. Of the TNBC subtypes, the IM and BLIA subtypes are classified as immune-rich.

CD8+ T cells are activated by antigen-presenting cells that present neoantigens on major histocompatibility complex class I and II [21]. They are directly activated by their receptor and further regulated by a complex interplay of co-stimulatory and co-inhibitory signals, known as immune checkpoints [8].

Through these mechanisms, tumors can hijack physiological immune responses and cause immune tolerance. Sequential clinical trials on the treatment of TNBC with anti-PD-1/PD-L1 monoclonal antibodies (pembrolizumab and atezolizumab), significantly increased overall survival rates. In 2019, the U.S. FDA approved the use of nab-paclitaxel in combination with atezolizumab for PD-L1+ TNBC. At the moment, the primary challenge is to improve the response of patients with TNBC to anti‒PD-1/PD-L1 treatment and to convert non-responders into responders.

## What about the Microbiome?

**Fig. 1.**
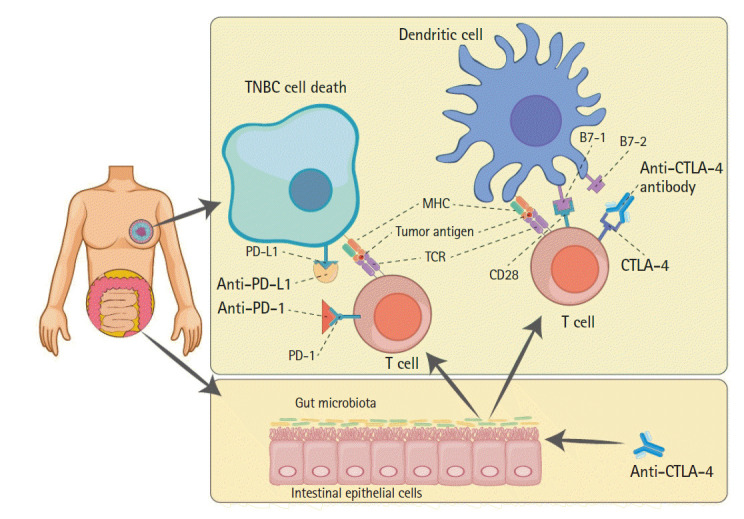
Schematic relations among ICB therapies and gut microbiota. The well-established anticancer mechanisms of anti–PD-1, anti–PD-L1, and anti-CTLA4 antibodies (bold text), as well-known ICB therapies, are illustrated; the efficacy of these therapies can be augmented by gut microbiota. The cytotoxic T-cell recognizing tumor antigen (red circle) causes TNBC cell death by blocking calm-down using ICBs. Gut microbiota metabolites can strengthen the anticancer effects of anti–CTLA-4 antibody and present the tumor antigen to T-cells interacting with dendritic cells (upper right). ICB, immune checkpoint blockade; PD-1, programmed cell death protein 1; PD-L1, programmed death-ligand 1; CTLA4, cytotoxic T-lymphocyte-associated antigen 4; TNBC, triple-negative breast cancer; TCR, T-cell receptor.

Strong evidence from recent studies has suggested that the gut microbiota can affect the host’s antitumor immunity and that the composition of the intestinal microbiome may modulate the efficacy of ICB therapy in mice and humans [22-28]. Previous studies revealed that specific components of the gut microbiome might influence the efficacy of ICB therapy in patients, and primary resistance to ICB therapy could be overcome through treatment with ICB-promoting bacteria [29-33]. Notably, Mager et al. [30] demonstrated that *Bifidobacterium pseudolongum*, isolated from ICB-treated tumors, modulated enhanced ICB effects through production of the soluble metabolite inosine. Elevated systemic inosine levels and activated antitumor T cells are caused by the induction of decreased gut barrier function, resulting from ICB therapy in colorectal adenocarcinoma, urothelial carcinoma, and melanoma mice models. Inosine binding to T cell-specific adenosine 2A receptor (A_2A_R) promotes Th1 cell activation [30]. Previous studies have demonstrated that inosine and A_2A_R binding exert an inhibitory effect on Th1 differentiation *in vitro* and antitumor immunity *in vivo* [34-36]. Emerging studies have implicated the involvement of the gut microbiome in response to breast cancer treatment. Recently, in 2018, Banerjee et al. [22] discovered a predominant microbial signature in TNBC, which includes the families *Caulobacteriaceae, Actinomycetaceae, Enterobacteriaceae, Prevotellaceae, Sphingobacteriaceae, Brucellaceae, Flavobacteriaceae*, and *Bacilliaceae*. In conclusion, some types of gut microbiota and their metabolites are available to develop microbial-based adjuvant therapies that enhance the effectiveness of ICB therapy in TNBC patients (Fig. 1).

## Conclusion

TNBC is the most aggressive type of breast cancer and has a poor prognosis. Because it lacks the expression of hormone receptors and HER2 receptors, therapy is primarily based on systemic chemotherapy, rather than targeted agents. Although TNBC has a higher mutational load than other breast cancer types, it generally responds well to ICB therapy. This led to FDA approval for the use of nab-paclitaxel in combination with atezolizumab in TNBC patients. Thanks to new promising treatments such as ICB monotherapy and ICB therapy combined with conventional systemic chemotherapy, we currently face exciting new perspectives. As discussed above, many efforts are being made to increase the antitumor effects of ICB, and studies are intensively investigating the individual gut microbiota. Collectively, this research program will provide a profound understanding of TNBC pathology and insights into antitumor mechanisms, the first step in developing therapeutic strategies for this devastating type of breast cancer.

## Figures and Tables

**Table 1.** Summary of the molecular classification of triple-negative breast cancer by gene expression

Variable	Lehmann et al. (2011) [9]	Burstein et al. (2015) [16]	Liu et al. (2016) [17]
No. of cases	587 (21 public datasets)	198	165
Subtype/Dysregulated pathway	6 subtypes	4 subtypes	4 subtypes
Cell cycle	BL1	BLIS	BLIS
DNA repair	BL2		
Immune signaling	IM	BLIA	IM
EMT signaling	M	MES	ML
Stemness-related signaling	MSL		
Hormone-related signaling	LAR	LAR	LAR
Modality	Gene expression profile	Gene expression profile copy number variation	Gene expression profile (mRNA+lncRNA)

BL1, basal-like 1; BLIS, basal-like immune-suppressed; BL2, basal-like 2; IM, immunomodulatory; BLIA, basal-like immune-activated; M, mesenchymal; MES, mesenchymal; ML, mesenchymal-like; MSL, mesenchymal stem-like; LAR, luminal androgen receptor; lncRNA, long noncoding RNA.
